# Supplementary material for: Applying the Health Stigma and Discrimination Framework to psychosis stigma in Malawi
Source: PLOS Ment Health. 2025 May 6;2(5):e0000306. doi: 10.1371/journal.pmen.0000306 (PMC12180428; doi:10.1371/journal.pmen.0000306)
Supplement: Attachment — Submitted filename: Response to Reviewers ENHANCE Stigma Study.docx [file pmen.0000306.s002.docx]

*Dear Editors,*

*We greatly appreciate your thoughtful reviews. We have responded to the comments as requested and made all changes to the manuscript in tracked changes. Our detailed responses to your comments can be found below each comment in italics.*

*Sincerely,*

*Authors*

Reviewer #1: Thank you for the opportunity to review this excellent paper. I have a couple suggestions for clarity and enrichment of your discussion but overall, feel it is a very interesting and important contribution to global mental health care

Introduction -
Your definitions of stigma are robust and draw on the work of Link & Phelan (and others) who are known scholars in this field. Given the allusion to Goffman's 'deeply discrediting attributes', I would suggest drawing on his work as well, as the seminal scholar on stigma.

*We thank the reviewer for this suggestion. We added the following context to the introduction expand the definition of stigma and cite Goffman’s seminal work: “Those with psychotic disorders are often discredited and labeled as “lesser” due to their behavioral symptoms.^1^* *This reduced social status is a result of the tendency to attribute behaviors to one’s internal characteristics rather than situational circumstances—a phenomenon that is common in the stigmatization process for mental disorders.^1,2^* *In Malawi, people often make this attribution error, attributing behavioral symptoms of psychosis to perceived faults such as the utilization of witchcraft or substance use.”*

The definition / elucidation of your chosen framework is fairly brief - it is novel, and a good application - I would recommend a little more detail here of the framework and your rationale for using it.

*We thank the reviewer for this recommendation. We added the following to description of the Health Stigma and Discrimination Framework to further describe how it is used and why it was chosen for this particular study: “This framework delineates the stigmatization process in the context of health, exploring the drivers and facilitators of health-related stigma, the manifestations of this stigma, and the impact of stigma on the individual-, interpersonal-, organizational-, community-, and public policy-levels.^5^ Recognizing the cultural and contextual nuances, the Health Stigma and Discrimination Framework provides a model for the universal process of stigmatizing health conditions, making it an ideal framework for this study.”*

Data collection -
Your methodology appears confined to a single opening sentence here. For a qualitative approach, please expand on the constructivist framework and its relevance / influence on the research. Which theorists did you draw on?

*We thank the reviewer for this comment. We have amended the text as follows:* “*The research team developed semi-structured qualitative guides based in a constructivist epistemology and formative research methodologies,^34^ with the overarching goal of gathering data to adapt a community-based rehabilitation intervention. Congruent with constructivists principles, the guides used open-ended prompts to elicit how participants consider, interpret and make meaning out of their experience with psychosis, with a particular focus on developing a deeper understanding of how individuals in Malawi conceptualize their illness, rehabilitation and recovery. In alignment with the study objective, the guides were organized as follows: 1) understanding of and experiences with psychosis; 2) care seeking and pathways to care; 3) perceptions of and experiences with stigma; 4) treatment, rehabilitation and goal setting; and 5) preferences for community-based rehabilitation care.”*

Although not a requirement for rigor or trustworthiness, there feels to be a missed opportunity in your results/ discussion exploring the relationship between mental health care and the persisting community perceptions around witchcraft and spirituality as drivers in mental illness - a more detailed context of how MH care is enacted in this context would add depth to the descriptions of stigma. Perhaps a brief historical overview of those community beliefs, or a sense of how healthcare professional in Malawi mediate between care and community beliefs?

*We appreciate this suggestion by the reviewer as we too believe understanding the impact of community beliefs on healthcare for people living with psychosis is a crucial aspect of this study. We added in some context to better explain how healthcare professionals may mediate between healthcare and community beliefs when caring for this patient population. We believe that this addition, included in quotations below, contributes to the context provided surrounding witchcraft and spirituality as it specifically references negative beliefs and prejudicial attitudes that are driven by fear and a lack of clinical psychiatric knowledge or training.*

*The following sentences were added to the Discussion section of this paper: “**Participants in this study described how experienced, anticipated and perceived stigma from society could lead to the internalization of stigma, a common occurrence among people with mental health disorders in low-resource settings.^8^ Stigma, in particular internalized stigma, has been linked to anxiety and depression, worse engagement in mental healthcare, more severe psychiatric symptoms, poor recovery, and a lower quality of life as it severely impacts the ability to perform daily activities and carry out roles that matter to the individual.^42,53–578^* *These negative health outcomes are further complicated by the cultural convention to seek traditional medical care before resorting to biomedical interventions.^9^ The resulting delay in accessing biomedical care may exacerbate symptoms and lengthen the duration of untreated psychosis,^10,11^ and contributing to psychosis stigma within healthcare facilities. Healthcare facility staff may interact with a higher caseload of patients with severe psychotic symptoms,^7^ and which may impact their attitudes towards people with psychosis and have implications for diagnostic overshadowing and delayed diagnosis and treatment.^58,59^ Specifically in the healthcare settings, participants highlighted the ways in which community stigma infiltrates psychosis care as many healthcare workers, particularly non-psychiatric and non-clinical staff, fail to reconcile community and personal beliefs regarding mental illness and their duty to provide high-quality care to PWLE. When presented with severe cases of psychosis, healthcare workers often held negative beliefs and prejudicial attitudes toward PWLE, likely due to fear and a lack of clinical psychiatric knowledge.”*

Thank you again - a pleasure to review and such interesting research.

Reviewer #2: Important piece of research in the field of stigma and discrimination and Psychoses.

1. Abstract – Add “developed semi-structured qualitative guides based in a constructivist epistemology and formative research methodologies” to the methods section

*We thank the reviewer for this recommendation as it provides further detail of our methodology in the abstract. The first sentence of methods section of the abstract now reads: “We developed semi-structured qualitative guides based in a constructivist epistemology and formative research methodologies and conducted 36 in-depth interviews (IDIs) and two focus-group discussions (FGDs) with 12 people with lived experience (PWLE) with psychosis; 12 caregivers of PWLE; six traditional healers; six medical providers; six community leaders (1 FGD); and six religious leaders (1 FGD) in Blantyre, Malawi.”*

2. Introduction
a. Any qualitative study requires a reading of the context to understand the underlying mechanisms of the process of stigma and discrimination. The authors need to describe the context of Malawi in this section.

*We thank the reviewer for this suggestion. We added a more specific contextual description of stigma and discrimination in Malawi following the addition of Goffman’s conceptualization of stigma as suggested by Reviewer #1. The following sentence was added to the introduction: “In Malawi, people often make this attribution error, attributing one’s behavioral symptoms of psychosis to perceived faults such as the utilization of witchcraft or substance use*.”

b. It will be useful to elaborate the reasons for choosing the Health Stigma and Discrimination framework – Page 8 lines 99-103

*We appreciate this feedback from the reviewer. In accordance with your feedback and Reviewer #1’s similar suggestion, we added the following description of the Health Stigma and Discrimination framework to further explain why this specific framework was chosen for this study: “This framework delineates the stigmatization process in the context of health, exploring the drivers and facilitators of health-related stigma, the manifestations of this stigma, and the impact of stigma on the individual-, interpersonal-, organizational-, community-, and public policy-levels.^5^ Recognizing the cultural and contextual nuances, the Health Stigma and Discrimination Framework provides a model for the universal process of stigmatizing health conditions, making it an ideal framework for this study.*”

3. Sample recruited
a. What was the duration of illness of those with Psychoses? Was everyone under treatment at the time of recruitment?

*Participants were asked about when they first began experiencing psychosis symptoms during the interviews. Not all participants were able to provide a clear reference timepoint. We’ve added the following to the text to describe: “The average time since first onset of psychosis symptoms was 11.6 years (Range 7-8).”*

*Everyone was in treatment at the time. We’ve amended the text of the methods as follows: “*(*1) people with lived experience (PWLE) with psychosis (confirmed by clinician diagnosis) receiving outpatient psychiatric care;”*

b. What was the relationship of the family caregivers of PWLE?

*We thank the review for this question and chance to clarify. The majority of caregivers were either parents or siblings of the PWLE. For clarification, we added the following sentence under “Study Population and Recruitment” in the Results section: “Caregivers were most often parents or siblings of PWLE.”*

c. How was informed consent obtained by the researchers, especially when educational levels were low?

*We appreciate this question from the reviewer. If participants were illiterate, study team members would read the informed consent forms to the participant in their local language in the presence of a literate witness. If the participant understood the content in the informed consent form, as evidenced by responding to an IRB-approved comprehension checklist, the participant would consent by marking a thumb print on the consent form along with the literate witness’ signature.*

4. Results and the Framework figure are well presented. It will be helpful if the researches can document the frequency of responses. For example, how many of the recruited participants reported on fear?
Was there any response in the data set that did not fit the framework?
As an option – the authors may present a table depicting the components of the framework with quotes as examples for each component.

*We appreciate the reviewer’s concerns and suggestions, and respectfully feel that it would be inappropriate to include counts to quantify the frequency of our findings. As discussed in the literature (citations below), there is a tendency to over-rely on count data in qualitative research, where sample sizes are small and are designed not to power analysis, but to yield rich detail into a topic. We have specifically chosen to integrate illustrative quotes from a variety of participant types into the body of the paragraphs within the results, for analysis and interpretation, the recommended practice for reporting qualitative data. Where appropriate, we comment on differences across groups.*

- *Maxwell, Joseph A. "Using numbers in qualitative research." Qualitative inquiry 16.6 (2010): 475-482.*
- *Neale, Joanne, Peter Miller, and Robert West. "Reporting quantitative information in qualitative research: guidance for authors and reviewers." (2014).*  [***https://doi.org/10.1111/add.12408***](https://doi.org/10.1111/add.12408)

*With respect to your question about responses that did not fit the framework - the Health Stigma and Discrimination Framework provides a model for delineating the stigmatization process, recognizing nuances in culture, context and even health condition. As such, there were no responses related to stigma that could not be organized and presented within the larger framework. However, there were areas of the Health Stigma and Discrimination framework that were less well described in our data. For example, at the “outcomes” level of the original Health Stigma and Discrimination framework, few of our participants discussed laws and policies, media, law enforcement practices, access to justice advocacy, or social protections related to psychosis or stigma around psychosis or investigate intersectionality. The lack of data around these topics may be a due to a lack of specific prompts within our guides to focus on these topics. However, given the open-ended nature of our semi-structured guides, it is also possible that these concepts (laws, polices, media, etc.) are less important or meaningful within the experience of psychosis and psychosis stigma in this low-resource, sub-Saharan setting.*

*We have added the following to the limitations section: “Considering our application of the Health Stigma and Discrimination Framework, the IDIs and FGDs yielded limited data around the role of laws and policies, media, law enforcement practices, access to justice, advocacy, or social protections in the stigmatization process and failed to investigate intersectionality. With an eye for dismantling the underlying power structures that drive health inequity and inequality,12 further inquiry into these topics may be warranted.”*

5. Discussion is fairly well written.

The authors could refer to the work of Koschorke et all in Social Science and medicine on work done in another LMIC - India (Koschorke M et al, . Experiences of stigma and discrimination of people with schizophrenia in India. Soc Sci Med. 2014 Dec;123:149-59. doi: 10.1016/j.socscimed.2014.10.035. Epub 2014 Oct 18. PMID: 25462616; PMCID: PMC4259492.)

*We thank the reviewer for this suggestion. We have added Koschorke et al.’s study as a reference and utilized some of this study’s findings in the discussion section to elaborate further on internalized stigma in patients with mental health disorders. The section on internalized stigma in the discussion now reads: “Participants in this study described how experienced, anticipated and perceived stigma from society could lead to the internalization of stigma, a common occurrence among people with mental health disorders in low-resource settings.^8^ Stigma, in particular internalized stigma, has been linked to anxiety and depression, worse engagement in mental healthcare, more severe psychiatric symptoms, poor recovery, and a lower quality of life as it severely impacts the ability to perform daily activities and carry out roles that matter to the individual.”*

6. PLOS authors have the option to publish the peer review history of their article ([what does this mean?](https://track.editorialmanager.com/CL0/https:%2F%2Fjournals.plos.org%2Fmentalthealth%2Fs%2Feditorial-and-peer-review-process%23loc-peer-review-history/1/010f019345baf056-33c8ffbb-2393-4734-84f1-8b59c6fd0040-000000/MQNmtozZ9cvWfRn1xKmuEOkJraPmZAG5OTcIBb_igdc=186)). If published, this will include your full peer review and any attached files.


**Do you want your identity to be public for this peer review?** If you choose “no”, your identity will remain anonymous but your review may still be made public.


For information about this choice, including consent withdrawal, please see our [Privacy Policy](https://track.editorialmanager.com/CL0/https:%2F%2Fwww.plos.org%2Fprivacy-policy/1/010f019345baf056-33c8ffbb-2393-4734-84f1-8b59c6fd0040-000000/VpauAa5GGSv6basAHJ_hVzTtR2oMq5YsvKmqTr70Ark=186).

Reviewer #1: **Yes:** Dr Floraidh Rolf

Reviewer #2: No
